# DNA topoisomerases as molecular targets for anticancer drugs

**DOI:** 10.1080/14756366.2020.1821676

**Published:** 2020-09-25

**Authors:** Kamila Buzun, Anna Bielawska, Krzysztof Bielawski, Agnieszka Gornowicz

**Affiliations:** a Department of Biotechnology, Medical University of Bialystok, Bialystok, Poland; b Department of Synthesis and Technology of Drugs, Medical University of Bialystok, Bialystok, Poland

**Keywords:** DNA topoisomerases, topoisomerase inhibitors, cancer, anticancer activity, anticancer drugs

## Abstract

The significant role of topoisomerases in the control of DNA chain topology has been confirmed in numerous research conducted worldwide. The prevalence of these enzymes, as well as the key importance of topoisomerase in the proper functioning of cells, have made them the target of many scientific studies conducted all over the world. This article is a comprehensive review of knowledge about topoisomerases and their inhibitors collected over the years. Studies on the structure–activity relationship and molecular docking are one of the key elements driving drug development. In addition to information on molecular targets, this article contains details on the structure–activity relationship of described classes of compounds. Moreover, the work also includes details about the structure of the compounds that drive the mode of action of topoisomerase inhibitors. Finally, selected topoisomerases inhibitors at the stage of clinical trials and their potential application in the chemotherapy of various cancers are described.

## Introduction

In 1971 Jim Wang discovered first DNA topoisomerase I (the omega (ω) protein from *Escherichia coli*)^1^^,^^2^. At that time, the separation of the supercoiled and relaxed DNA was necessary to perform the activity test. To achieve this, the reaction products were run on a sucrose gradient and then the DNA contained in each fraction taken from the centrifugation tubes were collected and analysed^2^.

In 1975, for the first time, agarose gel electrophoresis was used to differentiate between various DNA topoisomers. The use of this method greatly facilitated work for DNA topologists^3^. For nearly 40 years, many topoisomerases have been discovered and characterised in all three domains of life (bacteria, eukarya and archaea). The first discovery, in 1976, was bacterial DNA gyrase^4^ and then in 1980, eukaryotic decatenase was found^5^.

DNA topoisomerases are a group of enzymes that control DNA topology. They are involved in many significant biological processes in all cells (e.g. DNA replication, transcription and recombination or chromosome condensation)^6^. These enzymes bind covalently to the DNA phosphorus group, split the DNA strand or strands and finally reunite them. According to their mechanism of action, there are two main types of topoisomerases: topoisomerases I (Top I) and topoisomerases II (Top II) divided into five subfamilies (see Table 1).

**Table 1.** Types and subfamilies of topoisomerases

Type of topoisomerase	Subfamily	Subunit structure	Domains of life
I	IA	Monomer Heterodimer—reverese gyrase isolated from *Methanopyrus kandleri*	Bacteria, Archaea, Eukarya
	IB	Monomer	Eukarya and some viruses
	IC	Monomer	Archaea (*Methanopyrus* genus)
II	IIA	Heterotetramer - prokaryotic Top IIA Homodimer – eukaryotic Top IIA	Eukarya, Bacteria
	IIB	Heterotetramer	Archaea, Bacteria

The presence of topoisomerases and their proper functioning is one of the key elements of most processes taking place in the cell. For most of the processes requiring access to the information stored in the DNA duplex, a permanent or temporary separation of the two strands of DNA is necessary (e.g. topoisomerases enable the release of replicated chromosomes before partitioning and cell division)^7^.

The use of cytostatic agents, which inhibit enzyme activity, leads to irreversible interruption of DNA strands (by a stable DNA-topoisomerase complex), which causes cell death. Increased topoisomerase activity observed in many cancers results in selective action of agents that are topoisomerase inhibitors^8^.

In the beginning, we would like to briefly describe the topoisomerases I and II and their mechanism of action. Then we will present and characterise the topoisomerases I and II inhibitors, in particular compounds in clinical trials.

## Type I topoisomerases

Primarily named ω protein, topoisomerase I was discovered by James C. Wang in the 1970s. He identified the first topoisomerase I in *Escherichia coli*^2^. Proteins belonging to the group of topoisomerases I were found in eukaryotic and prokaryotic organisms^9–12^. These enzymes are responsible for relaxing negatively supercoiled DNA and allow for catenation or decatenation of broken DNA^11^^,^^13^.

Topoisomerases I can be divided into three subfamilies: topoisomerases IA, topoisomerases IB and topoisomerases IC^14–16^. For all subtypes of topoisomerases I, changing the DNA topology by breaking the phosphodiester bond between DNA strands is based on the same general mechanism. The phosphoryl group of DNA is attacked by tyrosyl group of topoisomerase I. This creates a covalent bond between the tyrosyl group and one side of the broken DNA. At the same time, the free hydroxylated strand is released and rotated. The hydroxyl end of the free strand of DNA attacks the formed phosphotyrosine bond, rebuilds the phosphodiester bond between the two strands and releases the topoisomerase to the next catalytic cycle^1^^,^^17^.

In most cases, change the topology of DNA by type I topoisomerases do not require external energy (e.g. ATP hydrolysis). Reverse gyrase is the only enzyme in topoisomerase I subfamily which needs energy from ATP hydrolysis to introduce positively supercoiled DNA. Now, one of the main challenges is to identify the way how energy stored in the DNA is converted into protein changes in the course of the reaction. Knowing this mechanism will help to fully understand not only type I topoisomerases but also other enzymes belonging to this class^9^.

### Type IA topoisomerases

Topoisomerases IA (Top IA) are a subfamily of enzymes found in all three domains of life (bacteria, archaea and eukarya). The sequence analysis has shown that all topoisomerases IA are monomeric enzymes (the exception—reverse gyrase isolated from *Methanopyrus kandleri*) composed of two main parts: a core (molecular weight around 67 kDa) with all the conserved domains, especially those creating the active site of the enzyme, and a carboxyl-terminal domain, very diverse in size and sequence^9^^,^^10^.

Type IA enzymes are responsible for relaxation only negative supercoils in DNA and a single-stranded region in DNA is required for their activity. The mechanism of action of the topoisomerases IA known as “enzyme-bridged strand passage” was defined based on structural, biochemical and biophysical studies. To change DNA topology type IA enzyme causes splitting of a single strand of DNA. The enzyme attaches tyrosine to the DNA 5′-phosphoryl group with a covalent bond. Released 3′-OH end remains attached Top IA and cannot freely rotate. This result in creating a gap that allows the transport of another DNA strand. After passing the transported segment through the gap, both ends of broken DNA are religated^9^^,^^18^^,^^19^.

Examples of enzymes belonging to the Top IA subfamily are bacterial topoisomerase I, bacterial topoisomerase III, eukaryotic topoisomerase III and reverse gyrase^1^^,^^6^^,^^20^.

### Type IB topoisomerases

**Figure 1.**
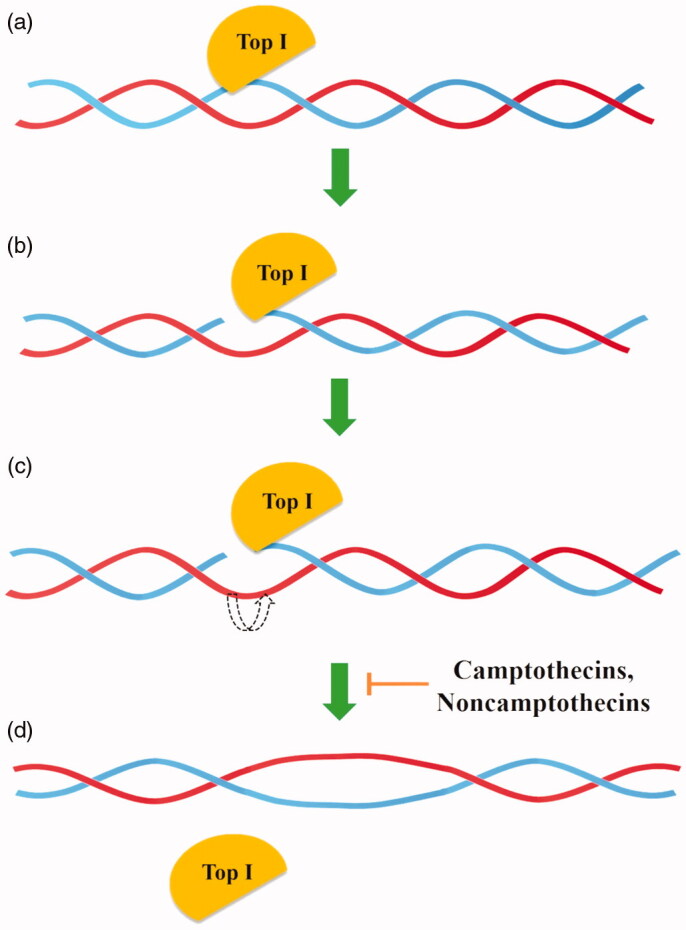
General mechanism of action of topoisomerase I (a) Top I binds to the DNA, (b) single-strand DNA (in blue) splitting, (c) controlled rotation of free DNA strand (in red), (d) religation of cleaved DNA strand.

Type IB topoisomerases (Top IB) are a subfamily of enzymes presented in eukaryotes, some viruses and bacteria but not in archaea^9^^,^^12^. As opposed to Top IA, topoisomerases IB can relax both negatively and positively supercoiled DNA with energy accumulated in DNA supercoils^12^. Type IB topoisomerases change DNA topology by “DNA rotation” based on the splitting of a single strand of DNA, covalent attachment of tyrosine to the 3′- phosphoryl group and release a free 5′-OH end. Temporary interruption of a single DNA strand enables the relaxation of supercoiled DNA by rotating the DNA molecule around the breakpoint. The reaction lasts until all torsional strain is removed and DNA is completely relaxed. Type IB enzymes do not require Mg^2+^ ions or ATP for relaxation of positive or negative supercoils in DNA (Figure 1)^9^^,^^21–23^.

Structures of all topoisomerases IB include a highly conserved pentad of residues creating the active site (Tyr, Arg, Arg, Lys and His/Asn). The only difference in the structure of this region between eukaryotic/viral (Tyr, Arg, Arg, Lys, His) and bacterial type IB enzymes (Tyr, Arg, Arg, Lys, Asn) is a replacement of histidine with asparagine in the bacterial pentad^9^.

The most important example of a type IB topoisomerases is the eukaryotic Top I (including human Top I), which is an important molecular target of anticancer therapies. In addition, Top IB group includes poxvirus topoisomerase and eubacterial Top IB^6^^,^^9^.

#### Human topoisomerase I

Human topoisomerase I (hTop I) belongs to Top IB subfamily. It is a monomeric enzyme and its molecular weight is 91 kDa^24^. The enzyme consists of 765 amino acids which form four domains: an N-terminal domain, a linker domain, a core domain and a C-terminal domain^25^. The detailed hTop I architecture was determined by X-ray crystallography^26^. There is a correlation between hTop I expression level and cell sensitivity to Top I inhibitors. While in healthy cells lower hTop I expression level has been observed, rapidly dividing cancer cells are observed to express a higher level of hTop I. Based on these results, we can assume that it is possible to target hTop I inhibitors towards cancer cells, tumours or patients with “overexpression” or increased expression of hTop I^24^.

### Type IC topoisomerases

Topoisomerase V (Top V) is a 110 kDa enzyme originally discovered in the archaeon *Methanopyrus kandleri*. So far, it is the only member of type IC topoisomerases subfamily^27–29^. Topoisomerase V is involved in DNA relaxation^27^ and DNA repair^30^^,^^31^. Similar to type IB topoisomerases, Top V can relax negatively and positively supercoiled DNA and cleaves single strand of DNA^13^. However, structural analyses showed a novel fold in the crystal structure of topoisomerase V and probably a different location of an active site of tyrosine. This allows us to suppose that the mechanism of DNA relaxation by Top V is different than in the case of type IB topoisomerases^29^. Based on that, topoisomerase V was classified as a member of new topoisomerase I subfamily named type IC topoisomerases^28^^,^^32^.

Topoisomerase I inhibitors used in the treatment of many types of cancer include compounds belong to a various class. These compounds will be presented and discussed in a separate chapter titled “Inhibitors of type I topoisomerases”.

## Type II topoisomerases

Type II topoisomerases, divided into two subfamilies (topoisomerases IIA and topoisomerases IIB) are presented in various organisms. Topoisomerases IIA (Top IIA) occur in bacteria, eukaryote and also in few species of archaea, whereas topoisomerases IIB (Top IIB) are present mainly in archaea, plants and certain algae. Based on structural and phylogenetic analyses, significant differences in global architecture and biochemistry between Top IIA and Top IIB were found^14^^,^^33^.

General mechanism of changing the topology of DNA by topoisomerases II is based on cleaving both strands of DNA duplex with Mg^2+^ and energy from ATP hydrolysis. Topoisomerase II covalently attaches tyrosine to the 5′ end of broken DNA, release a free 3′ end and allows to passing a second DNA duplex (the transported or T-segment) through a gap (the gate or G-segment)^1^^,^^33–35^. All enzymes belong to this family can relax both positive and negative supercoils in DNA^1^.

Due to their exceptional ability to untangle double strands of DNA, Top II is involved in many crucial nuclear processes, e.g. transcription, replication or recombination^36^^,^^37^. The formation of double-stranded DNA breaks as a result of a loss of activity by Top II leads to cell death. Moreover, disruption of the proper functioning of the enzyme by increasing the level of DNA cleavage (both genetic and drug-induced) leads to e.g. translocations of DNA^38^.

### Type IIA topoisomerases

Type IIA topoisomerases subfamily includes eukaryotic Top II, bacterial DNA gyrase, human topoisomerase II and bacterial Top IV^39^. The sequences of all the enzymes belonging to the family of type IIA topoisomerases (Top IIA) show significant similarity, and the only differences are found in the quaternary structures of these proteins. Gyrase and topoisomerase IV (Top IV) belonging to type IIA bacterial topoisomerases are composed of two subunits: the ParE and ParC which are homologues of the GyrB and GyrA subunits of gyrase. In terms of structure, prokaryotic type IIA topoisomerases are referred to as heterotetramers in contrast to eukaryotic enzymes which belong to the group of homodimers. The N-terminal half-ends of the eukaryotic Top IIA are homologues of the GyrB/ParE subunits of gyrase and Top IV, and the central parts of the enzymes are homologues of the GyrA/ParC subunits (gyrase/Top IV). The C-terminal half-ends, unlike the N-terminal parts of the eukaryotic topoisomerases type IIA, show significant structural differences between the enzymes of different eukaryotes. Analysis of C-terminal eukaryotic and prokaryotic type IIA topoisomerases did not show similarities in the sequence of these enzymes^40^.

**Figure 2.**
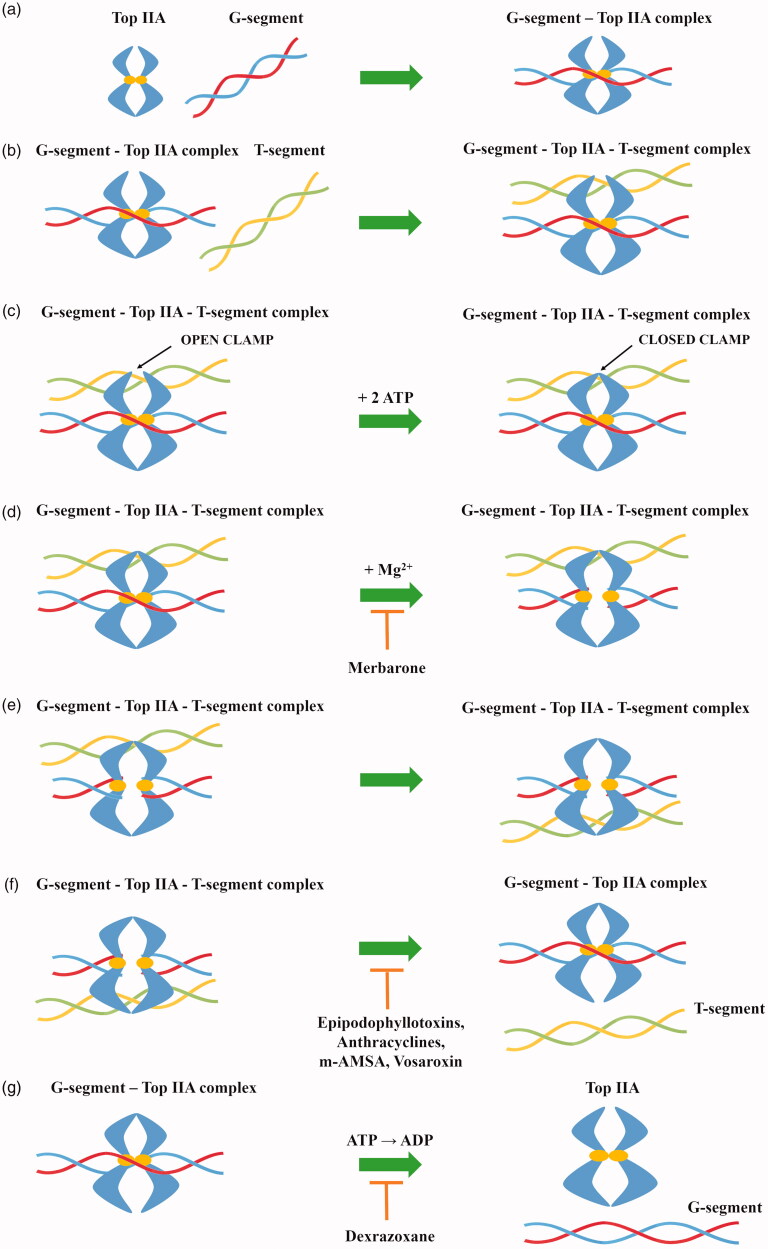
General mechanism of action of topoisomerase II (a) topoisomerase binds to the G-segment, (b) Top IIA- G-segment complex binds to T-segment, (c) Two ATP molecules are attached to the resulting complex, (d) G-segment cleavage in presence of Mg^2+^ ions, (e) T-segment transport through the created gap, (f) T-segment release and religation of G-segment broken strands and (g) hydrolysis of ATP molecules and release of the G-segment.

Type IIA topoisomerases perform DNA double-strand cleavage based on the “two-gate” mechanism (Figure 2)^41–43^. Initially, Top IIA binds to the first DNA helix (G-segment), which will be cleaved in the further stage of the catalytic cycle. In the next step, the resulting Top IIA- G-segment complex binds to the second DNA helix (T-segment) which will be transported through the created gap. Two ATP molecules are attached to the Top IIA—G-segment–T-segment complex causing conformational changes in the enzyme. As a result of hydrolysis of one ATP molecule to ADP, in the presence of Mg^2+^ ions, tyrosine from both Top IIA monomers attacks the phosphodiester bond of the first DNA helix resulting in cleavage of the strand with a shift of 4-bp and becomes covalently attached to the 5′ ends of the cleaved DNA (G-segment). Thanks to the transition, it is possible to transport the T-segment through a gap resulting after breaking the G-segment. Due to the hydrolysis of the second ATP molecule, the broken strands of the G-segment are religated. After passing through the gap, the T-segment is released. Then, due to the release of ADP molecules, the DNA-topoisomerase IIA complex is transformed from a closed into an open clamp, which is connected with the release of G-segment. Top IIA is free and ready for the next enzymatic reaction^44–47^.

#### Human topoisomerase II

Human topoisomerase II (hTop II) is composed of four domains: two N-terminal domains, a core domain and C-terminal domain. There are two homodimeric isoforms of hTop II: α (molecular weight around 170 kDa) and β (molecular weight around 180 kDa)^48^^,^^49^. Their amino acid sequences are homologous in approximately 70%^50^. Depending on the phases of the cell cycle, changes in human topoisomerase IIα (hTop IIα) and human topoisomerase IIβ (hTop IIβ) expression levels are observed. While the increased expression of hTop IIα is mainly observed in proliferating cells (the peak of expression is observed in G_2_-M phases), in case of hTop IIβ level of enzyme expression is constant regardless of the cell cycle stage^51^^,^^52^. hTop IIα is crucial for cell survival due to its role in chromosome condensation and segregation processes whereas hTop IIβ plays an important role in neurons development^53^^,^^54^.

### Type IIB topoisomerases

Commonly found in Archaea (except *Thermoplasmatales spp*.) type IIB topoisomerases were first discovered in the hyperthermophilic archaeon *Sulfolobus shibatae*^55^. The discovered enzyme was named topoisomerase VI (Top VI) and classified as a different subfamily of type II topoisomerases^56^. Like bacterial type IIA topoisomerases, Top VI is a heterotetramer enzyme composed of two subunits: A and B (A_2_B_2_). Structural and sequence analysis of type IIA and IIB topoisomerases did not reveal any similarities between the A subunits of both enzymes. The A subunit of Top VI shows similarity to eukaryotic Spo11 protein which is responsible for the initiation of meiotic DNA recombination as a result of the splitting of double DNA helix^57^. The B subunit of type IIB topoisomerases, responsible for binding and hydrolysis of ATP molecules shows significant structural similarity and slight sequence similarity to the B subunit of Top IIA^58^.

Among Archaea topoisomerase VI causes relaxation of positively and negatively supercoiled DNA and is also involved in DNA replication and transcription. Initiated by the type IIB topoisomerases changes in DNA topology by the formation of breaks in double-stranded DNA occurs with a shift of 2-bp rather than 4-bp as in type IIA topoisomerases^57^. Recently, topoisomerase VI has been identified in the plant kingdom^55^^,^^56^^,^^59^, including red and green algae^60^. Occurring in plants, Top VI plays an important role in the DNA endoreduplication process. Proper DNA endoreduplication is essential to maintain the correct size of plant cells and consequently the normal size of the entire plant^61^.

Scientific papers report the identification of new type IIB topoisomerase–topoisomerase VIII (Top VIII). It is the smallest known representative of type IIB subfamily of topoisomerases. The A and B subunits of this enzyme are merged into one single protein, hence the small size of Top VIII. Top VIII occurs in genomes of few bacteria species, two bacterial plasmids and one of the Archaea plasmids. In order to determine the structure and functions Top VIII, further detailed studies of this potential new type IIB topoisomerases subfamily member are necessary^62^.

## Topoisomerase inhibitors

Over the last few decades after discovering that doxorubicin induces DNA double helix cleavage via human topoisomerase II, scientific research based on searching of new compounds with an anticancer activity that are inhibitors of enzymes from the topoisomerases family has been intensified rapidly. To date, a significant number of topoisomerase inhibitors has been discovered and described in the scientific papers, however, in many cases, the detailed mechanism of action of these compounds is still unknown. Drugs classified as topoisomerase inhibitors can be divided into two groups: topoisomerase poisons or catalytic inhibitors. Doxorubicin, quinolones and many other anticancer or antibacterial drugs act as topoisomerases poisons. The mechanism of action of these compounds is based on the stabilisation of the formed enzyme–DNA covalent complex which prevents the religation of the cut strand^63^. The resulting DNA damages lead to cell death through apoptosis^64^. Catalytic inhibitors work by blocking the ability of the topoisomerase to attach to the substrate (DNA strand). Among this group there are two modes of action: the compound is binding to the DNA duplex or the topoisomerase. Regardless of the mode of action, catalytic inhibitors will lead to cell death through apoptosis^65^.

Tables 2 and 3 summarise information on important topoisomerase inhibitors and selected clinically investigated compounds, discussed in detail in this article.

**Table 2.** Clinically relevant topoisomerases inhibitors

Drug	Class	Molecular target	Date and country of approval	Application
Topotecan	Camptothecins	Top IB	1996–U.S.A.	Treatment of metastatic ovarian cancer, relapsed platinum-SCLC, recurrent or persistent cervical cancer
Irinotecan			1994–Japan	Treatment of colon cancer
Belotecan			2003–South Korea	Treatment of NSCLC and ovarian cancer
Etoposide	Epipodophyllotoxins	Top IIA	1983–U.S.A.	Treatment of SCLC, lymphomas (including non-Hodgkin’s lymphomas), AML, testicular and ovarian cancer
Teniposide			1992–U.S.A.	Treatment of childhood ALL, glioma, central nervous system tumours and bladder cancer
Doxorubicin	Anthracyclines		1974–U.S.A.	Treatment of various types of cancer i.a. ovarian, lung, gastric and breast cancers, multiple myeloma, thyroid cancer, Hodgkin’s and non-Hodgkin’s lymphoma, paediatric cancers and sarcoma
Epirubicin			1999–U.S.A.	Mainly in the treatment of advanced breast cancer
Valrubicin			1998–U.S.A.	Intravesical treatment of patients with BCG-refractory carcinoma *in situ*
Mitoxantrone	Anthracenediones		1987–U.S.A.	Treatment of acute leukaemia, lymphoma, prostate and breast cancer
Amsacrine	Acridines		1983–Canada	Treatment patients with AML and refractory ALL

**Table 3.** Selected topoisomerase inhibitors under clinical investigation

Compound	Study purpose	Clinical trial status	Clinical trial identification number
Namitecan	Determination of the pharmacokinetic profile and dose finding of the compound in treatment of patients with solid tumours	Phase I completed	NCT01748019
CZ-48	Examination the safety of CZ-48 administered orally	Recruitment of patients for the phase I trial	NCT02575638
AR-67	Application of AR-67 in the treatment of patients with refractory or metastatic solid malignancies	Phase I completed	NCT00389480
		Phase I completed	NCT01202370
	AR-67 application in the treatment of patients with recurrence of glioblastoma multiforme or gliosarcoma	Unknown	NCT01124539
Gimatecan	Safety, tolerance and pharmacokinetics study of the compound in fallopian tube cancer, advanced ovarian epithelial cancer or primary peritoneal cancer	Recruitment of patients for the phase I trial	NCT04029909
Edotecarin	Evaluatation the effectiveness of edotecarin with cisplatin in the treatment of advanced or metastatic solid tumours	Phase I completed	NCT00072332
	Determination of the effectiveness of treatment of women with chemoresistant locally advanced or metastatic breast cancer	Phase II completed	NCT00070031
LMP400	Application of the LMP400 and LMP776 in the treatment of adults with relapsed solid tumours and lymphomas	Phase I completed	NCT01051635
LMP776			
Genz-644282	Determination of the safety and tolerability of the compound	Phase I completed	NCT00942799
F14512	Evaluation of the maximum tolerated dose and the efficacy of combined therapy (F14512 and cytarabine) in patients (≥60 years old) with AML	Phase II completed	2012-005241-20 (EudraCT number)
Amrubicin	Application of amrubicin plus pembrolizumab in the treatment of refractory SCLC	Phase II is ongoing	NCT03253068
	The use of amrubicin for the treatment of relapsed or refractory thymic malignancies	Phase II completed	NCT01364727
	Potent application of the amrubicin in the therapy of HER2-negative metastatic breast cancer	Phase I completed	NCT01033032
Amrubicin	The use of amrubicin in combination therapy with cyclophosphamide for the treatment of advanced solid organ malignancies	Phase I completed	NCT00890955
Aldoxorubicin	Aldoxorubicin application in the treatment of glioblastoma	Phase II completed	NCT02014844
	The use of aldoxorubicin in the therapy of soft tissue sarcomas	Phase III completed	NCT02049905
	Application of combination therapy (aldoxorubicin plus gemcitabine) in the treatment of metastatic solid tumours	Phase I completed	NCT02235688
	Determination of the safety and efficacy of the standard chemotherapy in combination with aldoxorubicin and other drugs compared to standard chemotherapy in patients with locally advanced or metastatic pancreatic cancer	Recruitment of patients for the phase I trial	NCT04390399
Vosaroxin	Application of vosaroxin with azacitidine in treating older patients with AML	Recruitment of patients for the phase II trial	NCT03338348
	The use of vosaroxin in the treatment of patients with myelodysplastic syndromes	Phase I is ongoing	NCT01913951
Vosaroxin	Evaluation of combination therapy (vosaroxin with infusional cytarabine) in therapy of untreated AML	Phase II is ongoing	NCT02658487

### Inhibitors of type I topoisomerases

Mechanism of action of drugs classified as Top I inhibitors is based on the entrapment of created Top I-DNA covalent complex and prevents its cleavage^66–68^. This leads to permanent disruption of the DNA strands and consequently, to cell cycle arrest and induction of programmed cell death-apoptosis^69^^,^^70^.

#### Camptothecins (CPTs)

**Figure 3.**
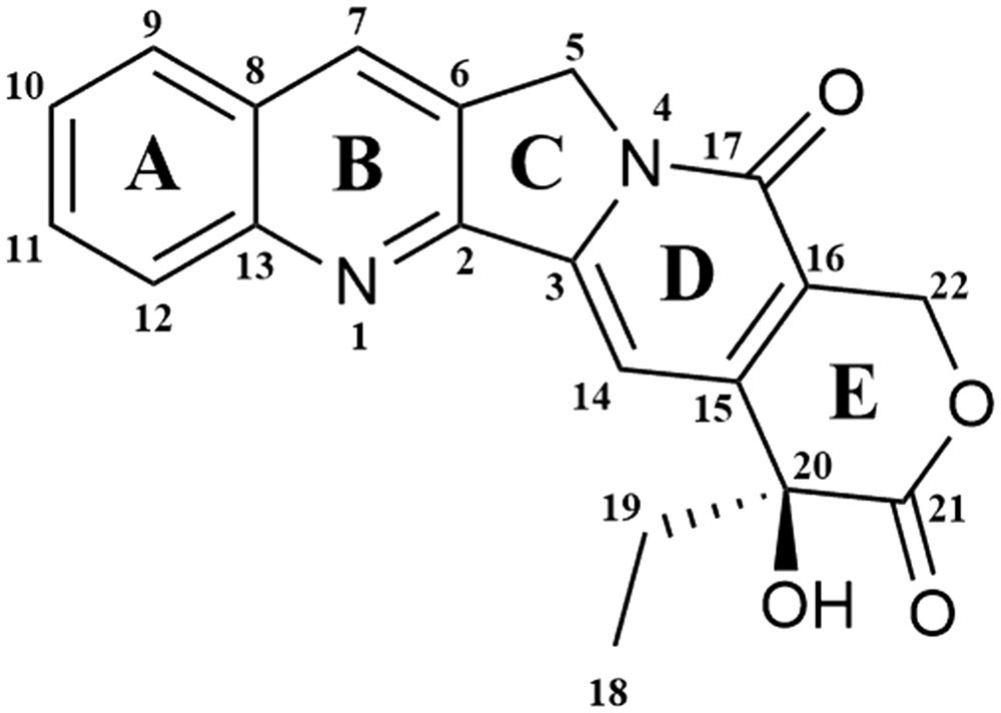
Chemical structure of camptothecin.

**Figure 4.**
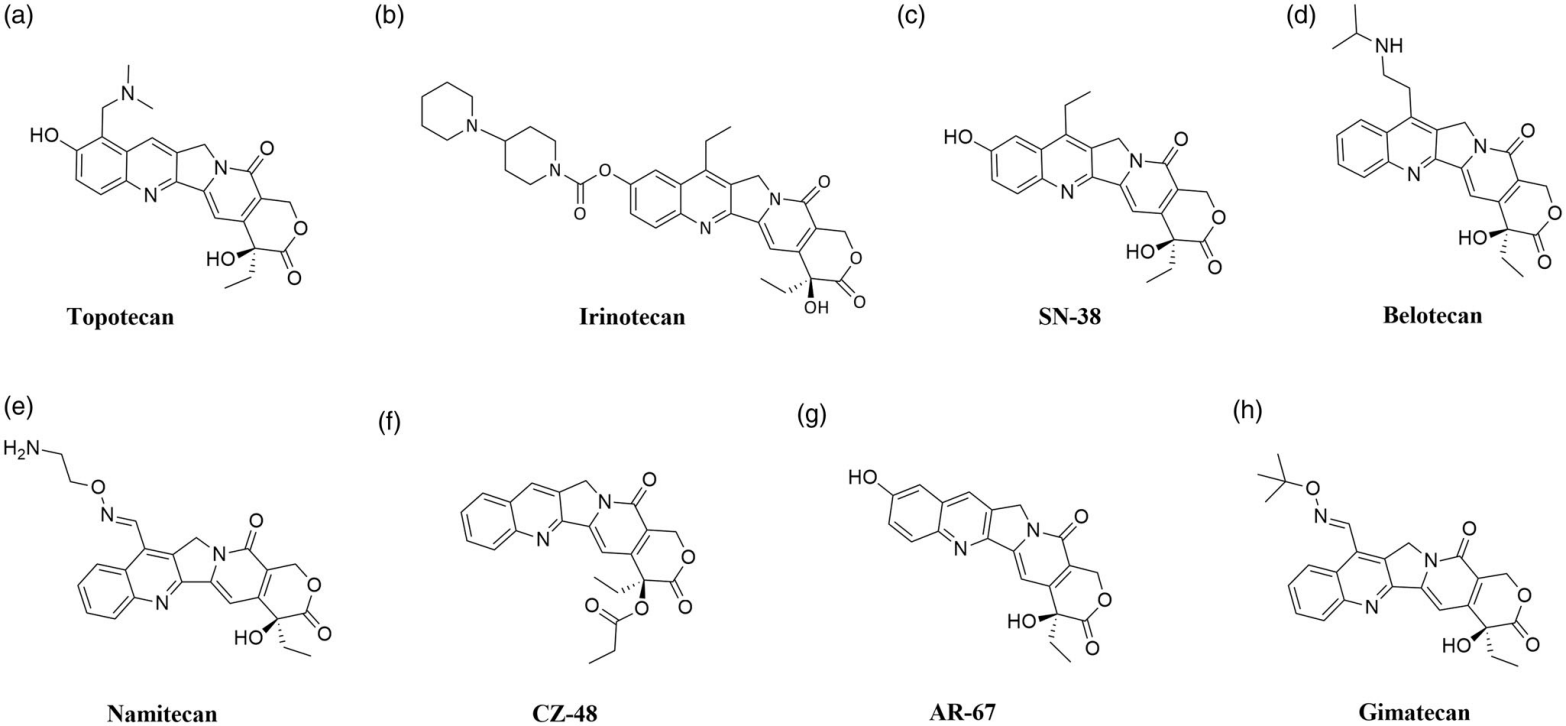
Chemical structures of camptothecins.

In 1985, it was confirmed that topoisomerase I is the molecular target of camptothecin anticancer activity^71^^,^^72^. Isolated in 1966 by Wall et al. from the bark of *Camptotheca acuminate* tree, camptothecin is a pentacyclic ring structure^73^. Structure–activity relationship studies revealed that modifications at C-7, C-9 or C-10 positions at quinolone moiety (A and B rings on Figure 3) increase the anticancer activity of CPTs^74^. The E-ring, interacting with topoisomerase at three sites, is the most important part of the CPTs structure. The − OH group located at C-20 position forms a hydrogen bond with the Asp-533 side chain in the Top I polypeptide chain, and Arg-364 of Top I binds with two hydrogen bonds to the lactone part of the CPTs E-ring. Moreover, the D-ring, responsible for the stabilisation of the covalent Top I–DNA complex, is hydrogen-bonded with +1 cytosine of the uncleaved DNA chain. This H bond is located between the amino group of the +1 cytosine pyrimidine ring and the CPTs carbonyl group on the D-ring C-17 position^75^. The molecular mechanism of action of the drug is based on reversible induction of single-strand breakages which affects the cell replication capacity. Top I-DNA cleavable complex is stabilised by camptothecin but the resulting breaks are non-lethal to the cell due to their full reversibility. The single-stranded breaks are transformed into irreversible double-stranded breaks as a result of the cleavable complex collision with DNA replication fork. Caspase activation leads to cell death by apoptosis^76^^,^^77^. As a result of the inhibition of caspase activation, the cells are temporarily arrested in the G_1_ cell cycle phase, which leads to necrosis^78^. S-phase-specific camptothecin shows its cytotoxic activity only when the DNA replication process is active. *In vitro* studies have shown that S-phase cells are 100–1000 times more sensitive to camptothecin than G_1_ or G_2_ cells^79^. Selected camptothecin derivatives used in the cancers treatment and compounds in the clinical trials (Figure 4) are discussed below.

#### Clinically important CPTs

Topotecan (9-[(dimethylamino)methyl]-10-hydroxycamptothecin; Hycamtin®) is semisynthetic, water-soluble camptothecin (CPT) derivative and it was the first CPT derivative approved in 1996 by the US Food and Drug Administration (FDA) for clinical use^80^. The studies conducted by Staker et al. allowed to determine the detailed mechanism of antitopoisomerase activity of topotecan (Figure 4(a)). DNA and compound intercalation occurs at the Top I-mediated DNA splitting site and is stabilised by interactions between the topotecan and −1 (upstream) and +1 (downstream) base pairs of DNA duplex. The rotation of the phosphodiester (0 P) bond results in the formation of an intercalation binding pocket. The movement of phosphodiester 0 P towards the binding pocket simultaneously stimulates energetically the release of +1 and −1 base pairs, which in turn is necessary for 0 P rotation. The transported 0 P phosphodiester is stabilised in the binding pocket as a result of the interaction between the two hydrogen bonds and the nitrogens of the main chain Gly-363 and Arg-362^81^. The understanding of the exact method of topotecan intercalation to DNA made it possible to explain the mechanism by which this compound blocks religation of the DNA strand. As a result of binding the topotecan molecule with the DNA, the +1 (downstream) base pair is shifted by 3.6 Å and a 5′-OH of the splitted DNA strand is shifted from the phosphotyrosine by 8 Å. Due to the fact that the topotecan binding pocket is located inside the DNA substrate and, moreover, is formed after the first transesterification, it becomes understandable why camptothecin and its derivatives interact with the Top I-DNA complex and not with the enzyme itself^82^. Topotecan, a known topoisomerase I inhibitor, has a broad spectrum of anticancer applications. It is successfully used for the second-line treatment of metastatic ovarian cancer in over 70 countries and the treatment of relapsed platinum-sensitive small-cell lung cancer (SCLC) in more than 30 countries around the world^83^^,^^84^. In 2006, topotecan was approved by the FDA for use to the treatment of recurrent or persistent cervical cancer not susceptible to surgery and/or radiation therapy^85^.

Irinotecan (7-ethyl-10-[4–(1-piperidino)-1-piperidino]carbonyloxycamptothecin; CPT-11) as topotecan is a water-soluble, semisynthetic camptothecin derivative (Figure 4(b)). It is a prodrug swiftly converted *in vivo* by human carboxylesterase 2 (hCE2) enzyme (occurs in liver, intestinal mucosa and plasma) to metabolite named SN-38 (7-ethyl-10-hydroxycamptothecin, Figure 4(c)) with high anticancer activity ^86^. hCE2 converts CPT-11 into its active metabolite SN-38 by hydrolysis and the metabolite shows a more than 100 times higher *in vitro* cytotoxicity than the parent compound^87^. The ionic strength, pH and protein concentration all influence the rate of hydrolysis of CPT-11 to SN-38. Both irinotecan and SN-38 occur in two forms - lactone and carboxylate. The lactone form with a closed *α*-hydroxy-*δ*-lactone ring is hydrolysed into a carboxylate form (open-ring hydroxyl acid). Based on the conducted research, it was found that the lactone form, whose antitumor activity is much more potent than the carboxylate form, is crucial for the stabilisation of the covalent Top I - DNA complex^88^. In liver, active SN-38 is metabolised into its inactive β-glucuronide form (SN-38G) by uridine-diphosphate glucuronosyltransferase (UGT)^89^. For the first time, irinotecan was approved in 1994 in Japan for the treatment of SCLC and non-small cell lung cancer (NSCLC), ovarian cancer and cervix cancer ^90^. Marketed by Pfizer Inc. and known as Camptosar® it is used for the treatment of colorectal cancer^91^.

#### Promising active CPT derivatives

Belotecan (7-[2-(N-isopropylamlino)ethyl]-(20S)-camptothecin; CKD-602, Camtobell®) is a relatively new, semisynthetic and water-soluble CPT derivative (Figure 4(d)). It was approved in 2003 in South Korea for the treatment of patients with NSCLC and ovarian cancer^92^. Belotecan exhibits similar efficacy profile compared to other CPT derivatives. However, its toxicity level is lower than the mentioned agents^93^. In 2018, Chong Kun Dang Pharmaceutical has completed the phase II clinical trial to evaluate the efficacy and safety of belotecan in comparison to topotecan in patients with relapsed SCLC (NCT01497873). Furthermore, in 2019 Lee et al. conducted *in vitro* and *in vivo* studies on the effects of CKD-602 in selected cervical cancer cell lines (CaSki, HeLa and SiHa). In CaSki-xenografted nude mice treated with CKD-602, a significant reduction of tumour volume was observed^94^.

Namitecan (7–(2-aminoethoxy)iminomethyl-camptothecin; ST1968) is one of the CPT derivatives (Figure 4(e)). Novel topoisomerase I inhibitor was evaluated in several preclinical studies with various tumour models (including resistant to topotecan/irinotecan xenografts, paediatric tumour models and diversified squamous-cell tumour models) and a significant antitumor activity was observed^95^. The conducted studies allowed us to determine the safety and pharmacokinetic profile of the namitecan. Furthermore, during phase I of clinical trials (NCT01748019), the anticancer activity of ST1968 in patients with endometrium and bladder cancer was observed^95^^,^^96^.

CZ-48 (camptothecin-20(S)-O-propionate hydrate) is CPT prodrug synthesised by Cao *et al*. in 2009. Researchers examined the anticancer activity of CZ-48 (Figure 4(f)) against 21 human tumour xenografts. Obtained results showed an excellent activity of the tested compound against various cancers (i. a. bladder, breast, colon, melanoma, lung and pancreatic tumour lines). In the case of 19 from 21 tested tumour lines, notable growth inhibition (>50%) or regression was observed^97^. Great anticancer activity and reduced toxicity compared to irinotecan and topotecan resulted in rapid clinical development of CZ-48. Cao Pharmaceuticals Inc. is currently recruiting patients with a solid tumour or malignant lymphoma of extranodal and/or solid organ site for phase I clinical trial to examine the safety of CZ-48 administered orally (NCT02575638).

AR-67 (7-*tert*-butyldimethylsilyl-10-hydroxycamptothecin; formerly DB-67) is a lipophilic CPT analogue synthesised by Bom *et al*. in the 1990s (Figure 4(g)). The scientist has designed a novel compound to improve blood stability and activity of CPT analogues. The conducted studies have shown *in vitro* and *in vivo* anticancer activity of the compound via interaction with topoisomerase I as well as improved stability of AR-67 in blood compared to clinically relevant CPTs^98^. The information provided by Arno Therapeutics and the University of Kentucky shows that phase I of the clinical trials on the potential application of AR-67 in the treatment of patients with refractory or metastatic solid malignancies (NCT00389480 and NCT01202370) has been completed. Furthermore, in 2014 Arno Therapeutics posted information about active phase II trial of AR-67 in patients with recurrence of glioblastoma multiforme or gliosarcoma (NCT01124539) however, the current study status remains unknown.

Gimatecan (7-*tert*-butoxyiminomethyl-camptothecin; ST1481) is novel, semisynthetic and lipophilic CPT analogue with good oral bioavailability (Figure 4(h)). Based on the promising results of the preclinical study, gimatecan was selected for clinical development^99^. Rapid absorption, compound accumulation and prolonged distribution in cancer cells are associated with Top I activity inhibition and stabilisation of the Top I DNA-Top I-gimatecan complex^100^. High efficacy of administered orally gimatecan against a panel of subcutaneous human tumour xenograft models, metastatic and orthotopic tumour models was observed^101^. Lee's Pharmaceutical Limited is currently recruiting patients with fallopian tube cancer, advanced ovarian epithelial cancer or primary peritoneal cancer for phase I clinical trial to investigate the safety, tolerability and pharmacokinetics of gimatecan (NCT04029909).

#### Noncamptothecins

**Figure 5.**
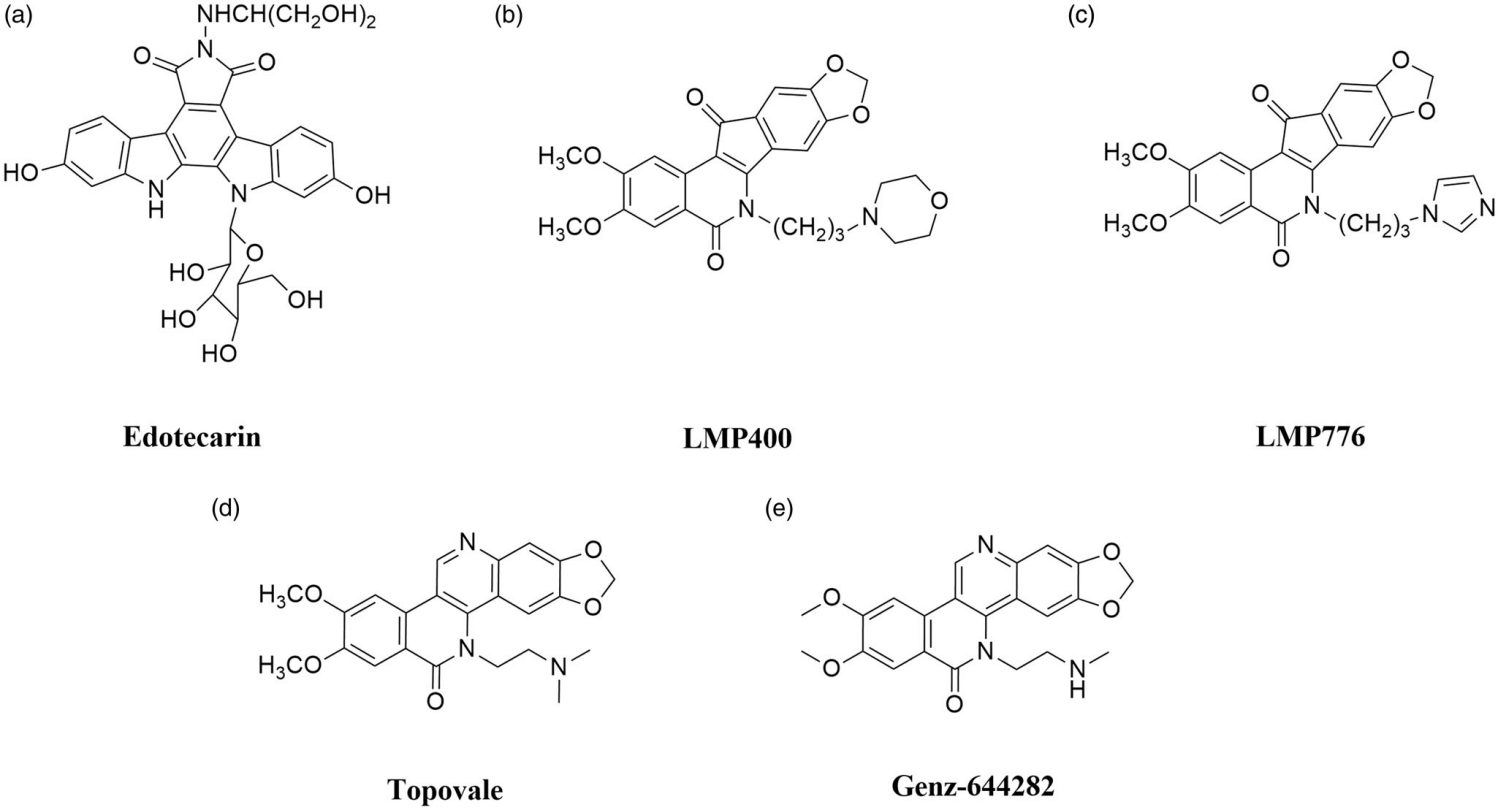
Chemical structures of noncamptothecins.

Nowadays, the only group of Top I inhibitors used in the oncological treatment are camptothecins. The excellent effectiveness of these compounds is accompanied by strong side effects (e.g. diarrhoea or neutropenia) and instability^6^^,^^20^. As mentioned above, camptothecins are characterised by chemical instability—compounds are immediately inactivated in the blood to carboxylate. Furthermore, due to the rapid reversal of Top I-DNA covalent complexes after drug removal, long infusions are necessary to ensure the effectiveness of treatment^102^. Described restrictions on the use of camptothecins have prompted scientists to investigate new noncamptothecin Top I inhibitors (Figure 5)^103^^,^^104^. Currently, in the clinical trials, there are compounds from indenoisoquinolines^105^, indolocarbazoles^106^ and dibenzonaphthyridinones^107^ groups.

#### Promising active noncamptothecins

##### Indolocarbazoles

Edotecarin (6-*N*-(1-hydroxymethyl-2-hydroxy)ethylamino-12,13-dihydro-2,10-dihydroxy-13-(*β*-D-glucopyranosyl)-5*H*-indolo[2,3-*a*]-pyrrolo[3,4-*c*]-carbazole-5,7(6*H*)-dione; J-107088) is a synthetic analogue of natural antibiotics isolated from several *Actinomycetes* (Figure 5(a)). It is a derivative of a compound named NB-506. Indolocarbazole NB-506 is an anticancer compound in which mechanism of antitumor activity is based on topoisomerase I or II poisoning. Edotecarin is a strong and specific topoisomerase I inhibitor, which is more effective in inducing single-strand DNA splitting compared to CPT or NB-506^108^. Furthermore, the stability of DNA-topoisomerase I covalent complexes formed as a result of edotecarin activity ensures longer effectiveness of the drug after the end of cell incubation with the compound^106^. Memorial Sloan Kettering Cancer Centre in collaboration with NCI conducted a phase I of clinical trials to evaluate the effectiveness of edotecarin combined with cisplatin in the treatment of patients with advanced or metastatic solid tumours (NCT00072332). In addition, the same collaborators completed phase II trial to study the effectiveness of treatment of women with chemoresistant locally advanced or metastatic breast cancer (NCT00070031).

##### Indenoisoquinolines

Indenoisoquinolines are developed in the 1990s by the NCI and represent a novel group of Top I inhibitors. These synthetic compounds are characterised by good chemical stability and better stabilisation of DNA–enzyme–drug covalent complexes than CPTs^103^^,^^109^. Among over 400 synthesised compounds, only two are currently under clinical trials: indotecan (LMP400; NSC 314622, Figure 5(b)) and indimitecan (LMP776; NSC 725776, Figure 5(c))^110^. Both compounds have been studied by scientists from the U.S. NCI in the treatment of adults with relapsed solid tumours and lymphomas (NCT01051635). Based on the information provided by the National Institutes of Health Clinical Centre, it is known that phase I of clinical trials has been completed^111^.

##### Dibenzonaphthyridinones

Next group of non-CPT topoisomerase I inhibitors are dibenzo[*c,h*][1,6]naphthyridinone derivatives. A novel group of compounds identified by LaVoie and his research team showed potent *in vitro* and *in vivo* anticancer activity^112^^,^^113^. Mechanism of topoisomerase inhibition by dibenzo[*c,h*][1,6]naphthyridinone derivatives is based on the stabilisation of cleavage DNA-Top I covalent complexes^114–116^.

Topovale (8,9-dimethoxy-5–(2-N,N-dimethylaminoethyl)-2,3-methylenedioxy-5*H*-dibenzo[*c,h*][1,6]naphthyridin-6-one; ARC-111) is a synthetic compound and strong Top I inhibitor (Figure 5(d)). The efficacy of topovale was evaluated against seven cancer cell lines and the obtained results showed higher cytotoxicity compared to the SN-38 or topotecan. Furthermore, the high effectiveness of this compound in cells which expressed the efflux pumps was observed^14^^,^^107^.

Next dibenzo[*c,h*][1,6]naphthyridinone derivative in clinical trials is Genz-644282 (8,9-dimethoxy-5–(2-*N*-methylaminoethyl)-2,3-methylenedioxy-5*H*-dibenzo[*c,h*][1,6]naphthyridin-6-one). The development of this drug was based on studies of structure-activity relationship in the family of dibenzo[*c,h*][1,6]-naphthyridin-6-one compounds^117^. Studies conducted to determine the cytotoxicity of Genz-644282 (Figure 5(e)) using bone marrow cells and cancer cell colony-forming assays have shown favourable cytotoxicity profile of this compound. Furthermore, based on the results obtained from studies of Genz-644282 activity in xenograft models, similar or higher activity of the tested drug was observed compared to the standard drugs^118^. Furthermore, in 2014 the phase I of the clinical trial on safety and tolerability of the compound in the therapy of solid tumours was completed (NCT00942799).

### Inhibitors of type II topoisomerases

The enzymes belonging to topoisomerases II family have the ability to achieve various conformational states as these proteins undergo significant structural changes during T-segment transport through the generated gate. In theory, trapping the enzyme in any conformational state allows manipulating of the topoisomerase II activity. To date, two types of Top II inhibitors have been identified and described: topoisomerase poisons and catalytic inhibitors^119^^,^^120^.

#### Topoisomerase poisons

Topoisomerase II poisons (Top II poisons) are the first from two types of Top II inhibitors. They cause inhibition of Top II ability to religation of cleaved DNA strands. Mechanism of action of Top II poisons is based on blocking of broken DNA ends rejoining as a result of slipping Top II poison molecules between splitted nitrogenous bases. This causes DNA double-strand breakage permanent by replicating broken DNA by cells. Moreover, replication of damaged DNA leads to the activation of the DNA damage response pathway and that in turn usually leads to cancer cell death through apoptotic pathway^121–123^.

##### Epipodophyllotoxins

For almost 2000 years, podophyllotoxins have been used in traditional medicine^124^. In the 19th century, podophyllin was identified as an effective drug used topically to treat skin cancers^125^. Despite the moderate anticancer properties of podophyllin and its derivatives selected in clinical trials, the high toxicity of tested drugs was observed^126^. As a result of research initiated in the 1950s by Sandoz Pharmaceuticals aimed at the synthesis of new derivatives with similar to podophyllin anticancer activity but significantly lower toxicity, in 1966 etoposide and one year later teniposide were synthesised^127^. Both podophyllin derivatives show a negligible affinity for DNA in the absence of Top II. Based on many studies, scientists have unequivocally concluded that drug–enzyme interactions are crucial for the proper functioning of drugs, and also indirectly participate in the formation of the Top II—DNA–compound ternary complex^128–130^.

#### Clinically important epipodophyllotoxins

**Figure 6.**
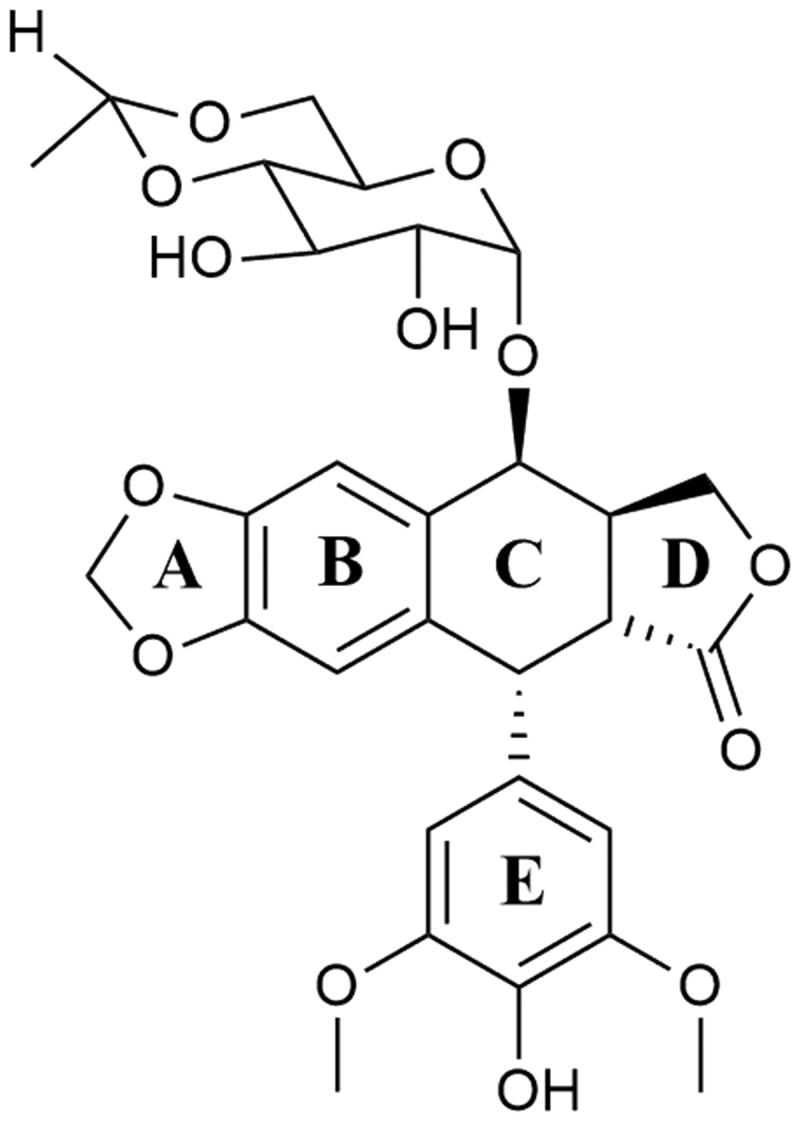
Chemical structure of etoposide.

Etoposide ((8a*R*,9*R*)-5-[(7,8-dihydroxy-2-methyl-4,4*a*,6,7,8,8*a*-hexahydropyrano[3,2-d][1,3]dioxin-6-yl)oxy]-9–(4-hydroxy-3,5-dimethoxyphenyl)-5*a*,6,8*a*,9-tetrahydro-5*H*-[2]benzofuro[6,5-f][1,3]benzodioxol-8-one; VP-16) is a semisynthetic podophyllotoxin derivative and clinically significant anticancer compound^124^^,^^131^. Discovered in 1966, VP-16 was approved by the FDA for cancer treatment in 1983^132^. The anticancer activity of etoposide is based on topoisomerase II poisoning by binding to the Top II-DNA covalent complexes^133^. This prevents religation of DNA strands, changing temporary DNA double-stranded breaks into permanent^134^, which indirectly leads to cell death by apoptosis. The combination of the results of the studies on the binary binding of the enzyme-drug complex, the research on DNA cleavage in the Top II-DNA-compound complex and NMR analysis made it possible to determine the functions of individual structures building the etoposide—the best-known epipodophyllotoxin. Presumably, the binding of etoposide (Figure 6) to Top II occurs through the interaction of the enzyme with the A- and B-ring of the compound, and also through the interaction between the enzyme and the E-ring of the compound. The − OH group and the methoxyl groups on the E-ring are an important element for the proper functioning of the drug. At the same time, both mentioned above groups do not significantly affect the specificity of DNA strands splitting or creating an enzyme-drug bond. The D-ring and the glycoside at position 4 of the C-ring probably interact with DNA found in the Top II—DNA–compound ternary complex, affecting the specificity of DNA cleavage. Interestingly, both structures do not interact with the enzyme found in the binary enzyme-drug complex^130^^,^^135^. Removal of the glycoside group from the compound’s structure does not significantly affect the etoposide-induced DNA cleavage process and substituting it with, for example, a spermine fragment (as in the case of the mentioned below compound - F14512) significantly increases the potency of the parent compound^136^. Etoposide is used for the treatment of SCLCs, lymphomas (including non-Hodgkin’s lymphomas), acute myeloid leukaemia (AML), testicular and ovarian cancer^123^^,^^137^^,^^138^.

**Figure 7.**
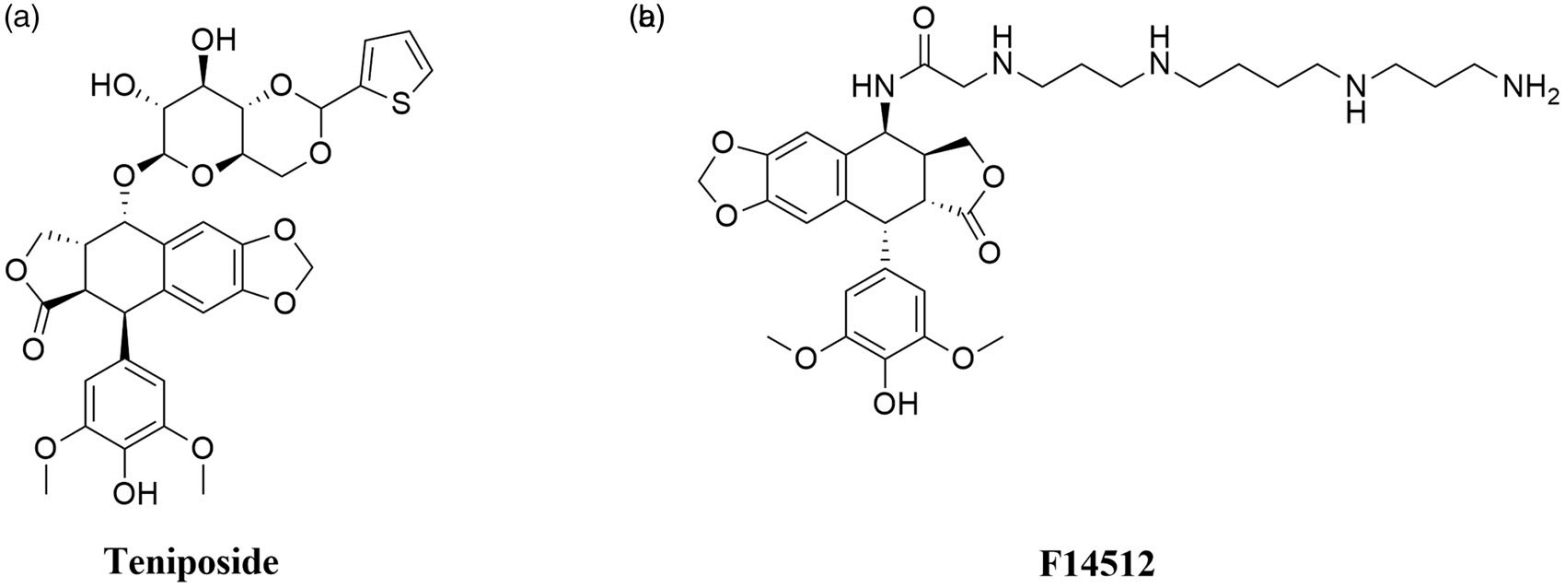
Chemical structures of epipodophyllotoxins.

Teniposide ((5*S*,5*aR*,8*aR*,9*R*)-5-[[(2*R*,4*aR*,6*R*,7*R*,8*R*,8*aS*)-7,8-dihydroxy-2-thiophen-2-yl-4,4*a*,6,7,8,8*a*-hexahydropyrano[3,2-d][1,3]dioxin-6-yl]oxy]-9–(4-hydroxy-3,5-dimethoxyphenyl)-5*a*,6,8*a*,9-tetrahydro-5*H*-[2]benzofuro[6,5-f][1,3]benzodioxol-8-one; VM- 26) is a second, semisynthetic derivative of podophyllotoxin and an analogue of etoposide (Figure 7(a)). Interestingly, VM-26 was discovered and tested in clinical trials before VP-16^124^^,^^127^. The mechanism of action of teniposide is similar to etoposide. It causes DNA double-stranded breaks via stabilisation of DNA-Topoisomerase II complexes^139^. Hypersensitivity reactions to teniposide in patients, as well as inappropriate administration of VM-26 using too low doses, caused slower development of the drug compared to etoposide. Consequently, teniposide was approved by the FDA in 1992, nearly 30 years after the first synthesis of the compound^140^. VM-26 is mainly used in the therapy of childhood acute lymphocytic leukaemia (ALL), glioma, central nervous system tumours and bladder cancer^141^^,^^142^.

#### Promising active epipodophyllotoxin derivatives

F14512 (*N*-[(5*S*,5*aS*,8*aR*,9*R*)-9–(4-hydroxy-3,5-dimethoxyphenyl)-8-oxo-5*a*,6,8*a*,9-tetrahydro-5*H*-[2]benzofuro[5,6-f][1,3]benzodioxol-5-yl]-2-[3-[4–(3-aminopropylamino)butylamino]propylamino]acetamide) is a novel etoposide derivative developed by Barret et al. The compound was designed by the replacement of VP-16 carbohydrate group with a polycation spermine moiety (Figure 7(b)). The modification of VP-16 structure resulted in a significant increase in compound solubility, cellular uptake, and cytotoxicity of F14512^136^. The presence of a spermine moiety in the compound molecule led to an increase in the degree of DNA binding and consequently to an enhancement of the inhibition of Top II activity^143^. Moreover, the overexpression of polyamine transport system observed in cancer tumours was used to intensify selective drug uptake by cancer cells^144^^,^^145^. *In vitro* activity of F14512 was evaluated in 29 human cancer cell lines (i.a. leukaemia, sarcoma, myeloma, prostate, pancreatic and ovarian cancer cell lines) and noteworthy cytotoxicity in 21 cell lines was observed^136^. F14512 *in vivo* anticancer activity was investigated using 19 experimental models. In 13 out of 19 investigated models, F14512 exhibited a strong antineoplastic activity^146^. Furthermore, based on the information provided by the EU Clinical Trials Register, it is known that phase I–II study to evaluate the maximum tolerated dose and the efficacy of combined therapy of F14512 and cytarabine in patients (≥60 years old) with AML has been completed (EudraCT number: 2012–005241-20).

##### Anthracyclines

Anthracyclines were isolated in the 1950s from *Streptomyces peucetius* which is one of the *Actinomyces* species. Obtained compounds showed antibacterial properties *in vitro*^147^. For over 50 years anthracyclines combined with various cytotoxic agents or targeted agents have been the most commonly used anticancer drugs to the treatment of solid or haematological tumours^148^^,^^149^. As topoisomerase poisons, anthracyclines stabilise DNA-topoisomerase II covalent complexes, which indirectly leads to apoptosis via double-stranded DNA breaks and inhibition of DNA transcription and replication processes^150^. A characteristic feature of all anthracycline derivatives is that the structures of individual compounds slightly differ from one another. These slight modifications cause significant changes in compounds activity. For instance, modification of the compound’s structure by removing the 3-amino or 4-methoxy substituent significantly increases the activity of the compound. Moreover, the presence and nature of the substituent at position 3 of the sugar moiety is crucial for the selectivity of the anthracyclines DNA cleavage process^151^.

#### Clinically important anthracyclines

**Figure 8.**
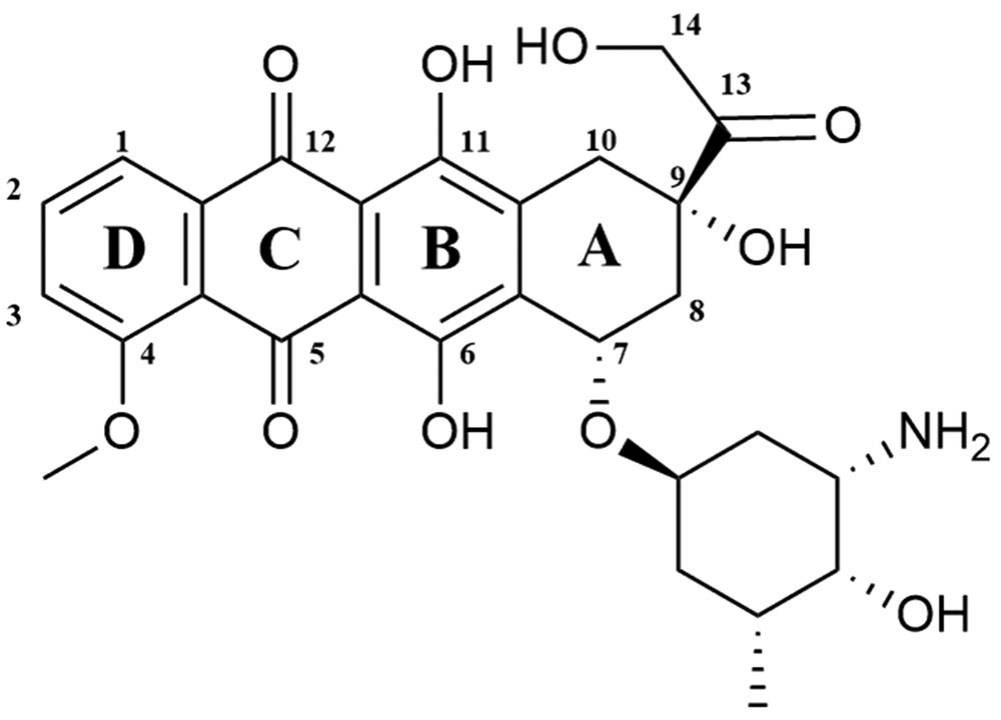
Chemical structures of doxorubicin.

Doxorubicin ((7*S*,9*S*)-7-[(2*R*,4*S*,5*S*,6*S*)-4-amino-5-hydroxy-6-methyloxan-2-yl]oxy-6,9,11-trihydroxy-9–(2-hydroxyacetyl)-4-methoxy-8,10-dihydro-7*H*-tetracene-5,12-dione; Adriamycin) is a chemotherapeutic drug which belongs to the anthracyclines group. It was first extracted in the 1970s from *Streptomyces peucetius var. caesius*^152^. The doxorubicin molecule is composed of sugar and aglycone moieties. The aglycone (doxorubicinone) consists of a tetracyclic ring linked to a hydroquinone and quinone residue, a short side-chain containing a primary alcohol at C-14 atom and a carbonyl group at C-13 position (Figure 8). The aminosugar (daunosamine) is linked by a glycosidic bond with the C-7 carbon located in the A-ring^153^. The molecular docking analyses enabled the researchers to identify the mechanism of interaction between the Top II–DNA complex and DOX. The obtained results showed that the planar DOX molecule is located between the GC base pairs of cleaved DNA strands. Three H bonds are formed between topoisomerase and DOX. The −OH group of Ser-740 Top II combines with the −OH group located in position C-11 in the anthraquinone fragment and Thr-744 forms a bond with DOX carbonyl oxygen located in position C-12. The last hydrogen bond is formed between the side chain Gln-750 and the C-14 atom of DOX^154^. A-ring and the DOX sugar moiety also interact with the DNA itself, forming H bonds between a base complement to the base at the −1 position in DNA and the −OH group in the C-9 position of the DOX A-ring. Scientists speculate that the interaction between C-9 of A-ring of DOX and DNA is crucial to stabilise the drug–molecular target complex. Proper recognition of DNA threads by compound may be associated with the formation of hydrogen bonds between DNA and daunosamine moiety. G-2 and C-1 of DNA bond with the −OH group in position 4′ and the amine group located in position 3′ of daunosamine, respectively^155^. There are two main mechanisms of action of doxorubicin (DOX) which lead to the inhibition of the catalytic cycle. At the beginning of the catalytic cycle, doxorubicin can interfere with DNA binding which prevents relaxation or (de)catenation of DNA by Top II. Furthermore, DOX can prevent the religation of G-segment cleaved strands^156^. Despite its anticancer properties doxorubicin is responsible for serious side effect which is dose-dependent cardiotoxicity^157^^,^^158^. The exact mechanism of DOX cardiac toxicity is still unclear. However, the most probable pathway is related to the formation of iron-related free radicals^159^^,^^160^. Strong evidence, supporting this hypothesis is the fact that dexrazoxane, a known iron chelator, is used as a doxorubicin-induced cardiotoxicity protector^152^^,^^161^. Approved by FDA in 1974, it is used in the therapy of various types of cancer i.a. ovarian, lung, gastric and breast cancers, multiple myeloma, thyroid cancer, Hodgkin’s and non-Hodgkin’s lymphoma, paediatric cancers and sarcoma^152^^,^^162^.

**Figure 9.**
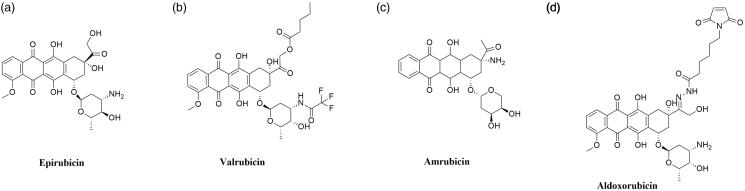
Chemical structure of anthracyclines.

Epirubicin ((7*S*,9*S*)-7-[(2*R*,4*S*,5*R*,6*S*)-4-amino-5-hydroxy-6-methyloxan-2-yl]oxy-6,9,11-trihydroxy-9–(2-hydroxyacetyl)-4-methoxy-8,10-dihydro-7*H*-tetracene-5,12-dione; 4′-epidoxorubicin) is anthracycline derivative (Figure 9(a)), epimer of doxorubicin^163^. Epirubicin (EPI) shows activity in all phases of the cell cycle, but the highest activity is observed in the S and G_2_ phases. The mechanism of action of EPI is similar to doxorubicin. The drug inhibits the topoisomerase II activity by stabilisation of DNA–topoisomerase II covalent complexes which prevent splitting of DNA strands^164^. In 1999 it was approved for clinical use in the United States. The anticancer activity of EPI was confirmed against a broad spectrum of cancers but it is mainly used against advanced breast cancer^165^.

Valrubicin (N-trifluoroacetyladriamycin-14-valerate; AD-32) is anthracycline derivative and Top II inhibitor obtained by modification of DOX (Figure 9(b)). Substitutions of two side chain of DOX resulted in the formation of a novel molecule with enhanced safety profile and lack skin toxicity, which allows the topical application of the compound^166^^,^^167^. The compound developed by Anthra Pharmaceuticals, Inc. (Valstar®) was approved by the FDA in 1998 for intravesical treatment of patients with BCG-refractory carcinoma *in situ*^168^. Currently ongoing clinical trials investigate the application of valrubicin i.a. in the treatment of early-stage bladder cancer (NCT00003129, phase II completed) and upper tract urothelial carcinoma (NCT01606345, phase I completed).

#### Promising active anthracycline derivatives

Amrubicin (9-amino-anthracycline; SM-5887) is a synthetic anthracycline derivative with potent antitumor activity based on the hTop II inhibition (Figure 9(c)). Cytotoxic activity of amrubicin results from the stabilisation of DNA–hTop II cleavable complexes^169^^,^^170^. Moreover, amrubicinol, an active amrubicin metabolite, is 5–220 times more cytotoxic than the original compound^171^. In Japan, amrubicin has been approved for the treatment of patients with NSCLC and SCLC^172^. Furthermore, it is used as a therapy option in the chemotherapy of NSCLC after the 3rd-line treatment^170^. Phase II of clinical trials for the use of amrubicin plus pembrolizumab in the treatment of refractory SCLC (NCT03253068) is currently ongoing. In addition, research concerning the use of amrubicin for the treatment of relapsed or refractory thymic malignancies (NCT01364727, phase II completed), HER2-negative metastatic breast cancer (NCT01033032, phase I completed) and in combination with cyclophosphamide for the treatment of advanced solid organ malignancies (NCT00890955, phase I completed) is being conducted.

Aldoxorubicin ((6-maleimidocaproyl)hydrazone of doxorubicin; formerly INNO-206) is doxorubicin prodrug developed by CytRx Corporation (Figure 9(d)). The aldoxorubicin (ALDOX) molecule consists of doxorubicin conjugated with a linker (6-maleimidocaproic acid hydrazide). After intravenous injection, ALDOX is quickly and selectively attached to cysteine-34, which belongs to the group of endogenous amino acids of serum albumin. After binding to albumin, DOX is transported to the tumour. The acidic tumour environment breaks the acid-labile hydrazine bond between the linker and the drug. The release of DOX, intercalation of the drug with DNA and inhibition of topoisomerase II activity occurs^173^^,^^174^. Preclinical studies performed on mouse xenograft tumour models of ovarian, breast, lung and pancreatic cancers have shown higher ALDOX anticancer activity compared to DOX^175^^,^^176^. Research conducted by Sanchez et al. demonstrated a significant *in vitro* and *in vivo* ALDOX activity against multiple myeloma cells. Moreover, an enhanced effect of combined ALDOX and bortezomib therapy of multiple myeloma compared to single-agent therapy (ALDOX or bortezomib alone) was observed^177^. Over the last years, numerous clinical trials on the use of ALDOX in the treatment of various cancers have been initiated. Research on the use of ALDOX in the treatment of glioblastoma (NCT02014844, phase II completed), soft tissue sarcomas (NCT02049905, phase III completed) as well as in combination with gemcitabine in the treatment of metastatic solid tumours (NCT02235688, phase I completed) is ongoing. Furthermore, in May 2020 ImmunityBio, Inc. reported the launch of a clinical trial to determine the safety and efficacy of the standard chemotherapy in combination with aldoxorubicin and other drugs compared to standard chemotherapy in patients with locally advanced or metastatic pancreatic cancer (NCT04390399).

##### Anthracenediones

**Figure 10.**
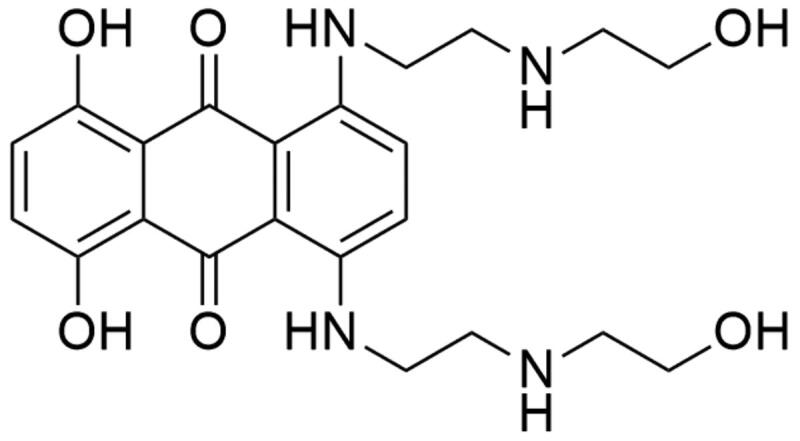
Chemical structure of mitoxantrone.

Mitoxantrone (1,4-dihydroxy-5,8-bis[2–(2-hydroxyethylamino)ethylamino]anthracene-9,10-dione; MTX) is a synthetic chemotherapeutic agent belonging to the anthracenedione derivatives (Figure 10)^178^. It was discovered in the 1970s by two independent groups of investigators: the Medical Research Division of the American Cyanamid^179^ and the Midwest Research Institute^180^. The search of new antineoplastic drugs began with a study of structure–activity relationships of new anthracenedione derivatives. Based on the obtained results, which revealed that the presence and composition of side-chains have a large impact on the activity of anthracenediones^179^^,^^181^ MTX was selected for further studies as a potent anticancer compound^182^. Conducted in the 1990s, footprinting studies revealed that mitoxantrone-mediated DNA cleavage occurs at sites having a thymine or cytosine residue in close proximity. Additionally, the location of DNA cleavage is influenced by the presence of guanine located two nucleotides downstream—in the +2 position^183^. X-ray crystallographic analysis enable scientists to build the 3 D model of Top II–DNA–MTX complex. A single drug molecule is introduced exactly at both sites of splitted DNA strands. The dihydroxyanthracenedione chromophore of the MTX molecule has a dual function—through intercalation, it docks the MTX molecule in the DNA duplex and allows direct contact between the additional hydrogen bonds and amino acid residues of the protein. Two hydroxyalkylamine side-chains of the MTX molecule “surround” nucleobases located vis-a-vis the DNA breakpoint. At the same time, such method of “surrounding” DNA allows interaction with closer amino acid residues of the protein, which are responsible for further stabilisation of the complex^184^. The anticancer activity of MTX is based on inhibition of topoisomerase II activity by stabilisation of DNA-topoisomerase II covalent complex, which leads to DNA breakages. Splitting of DNA strands results in inhibition of two significant processes: replication of DNA and transcription of RNA^185^^,^^186^. MTX was approved by the FDA in 1987 and nowadays it is the only clinically approved drug from the anthracenedione derivatives group^187^. It is used as a chemotherapeutic drug in the treatment of acute leukaemia, lymphoma, prostate cancer and breast cancer^188^. Furthermore, MTX is used in the treatment of multiple sclerosis^189^.

##### Acridines

**Figure 11.**
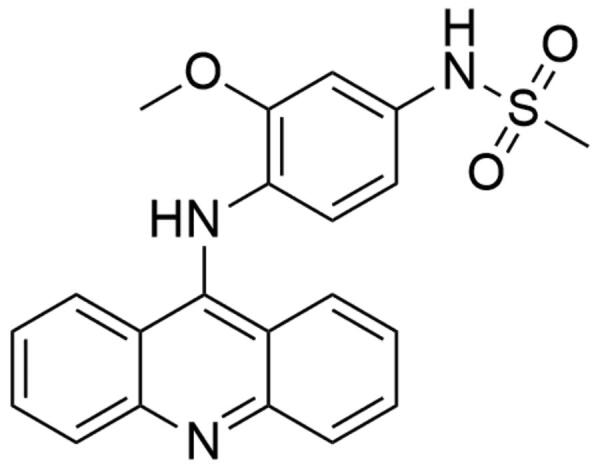
Chemical structure of amsacrine.

Amsacrine (*N*-[4-(acridin-9-ylamino)-3methoxyphenyl]methanesulfonamide; *m*-AMSA) is hTop IIβ inhibitor belonging to the class of acridines. The amsacrine molecule is composed of two basic elements: the acridine group and the 4′-amino-methanesulfone-*m*-anisidide head group (Figure 11). To determine the role of *m*-AMSA-mediated DNA binding in the proper functioning of the compound and to perform a comprehensive structure–activity relationship analysis, scientists analysed different amsacrine derivatives. The compounds were tested for their possibility of DNA intercalation and the ability to increase the intensity of the DNA strand cleavage process with the participation of hTop IIα and hTop IIβ. The obtained results indicate that the presence of the methoxy group in the 3′ position (*m*-AMSA) has a positive effect on the anti-tumour activity of the drug. In turn, changing the location of the group by placing it in the 2′ (*o*-AMSA) position causes inhibition of drug activity. It is probably caused by an increase in the freedom of rotation of the 4′-amino-methanesulfone-*m*-anisidide group, which may disrupt, e.g. the intercalation process of the 1′ substituent in the Top II–DNA–drug complex^190^. Moro et al. conducted modelling of *m-*AMSA interaction with the Top II–DNA complex. Based on the obtained results, it was found that the CG and GC base pairs of DNA, which form a clamp, are 3.4 − 6.8 Å away from each other. This arrangement of base pairs allows locating the drug molecule between them. The aniline sulphonamide in position 1′ strongly binds with –OH Thr-744 group. The oxygen of Gly-747 carbonyl group and NH of sulphonamide residue form another strong bond. Moreover, hydrophobic interaction was observed between –CH_3_ group in position 3′ of aniline methoxyl substituent and side-chain Phe-754^155^. The drug was approved in 1983 in Canada^25^ and to date is used for the treatment patients with AML and refractory ALL. The inhibitory effect of *m*-AMSA on hTop IIβ activity is based on the topoisomerase poisoning and increasing level of DNA- hTop IIβ covalent complexes in the cell^190^^,^^191^. Furthermore, few clinical trials are conducted to evaluate the effectiveness of combination therapy (*m*-AMSA with various compounds) in the treatment of several cancers (NCT03765541, NCT00003436, NCT00002719).

##### Quinolones

**Figure 12.**
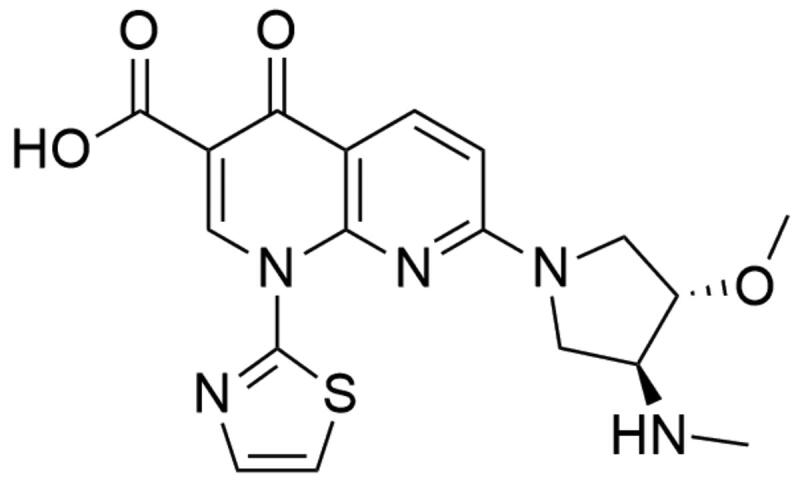
Chemical structure of vosaroxin.

Vosaroxin (7-[(3*S*,4*S*)-3-methoxy-4-(methylamino)pyrrolidin-1-yl]-4-oxo-1–(1,3-thiazol-2-yl)-1,8-naphthyridine-3-carboxylic acid; formerly AG-7532, SN-595 or voreloxin) was first described in 2002. It is a synthetic, antineoplastic quinolone derivative (Figure 12) developed by Sunesis Pharmaceuticals^192^^,^^193^. It is the first anticancer quinolone derivative and its antineoplastic mechanism of action is based on the topoisomerase II poisoning and induction of site-specific breaks in double-stranded DNA^194^. Currently, there are ongoing clinical studies concerning the application of vosaroxin with azacitidine in treating older patients with AML (NCT03338348, University of Ulm is recruiting participants for the phase II trial). Phase I trial in the treatment of patients with myelodysplastic syndromes (NCT01913951) is ongoing. Moreover, combination therapy (vosaroxin with infusional cytarabine) is evaluated in untreated AML therapy (NCT02658487, phase II trial is ongoing).

#### Catalytic inhibitors

Catalytic inhibitors are the second from two types of Top II inhibitors. Their mechanism of action is based on preventing the DNA-Top IIA bond formation, stabilisation of DNA by the formation of non-covalent complexes with Top IIA or blocking the ATPase enzyme binding site^195–197^.

##### Bisdioxopiperazines

**Figure 13.**
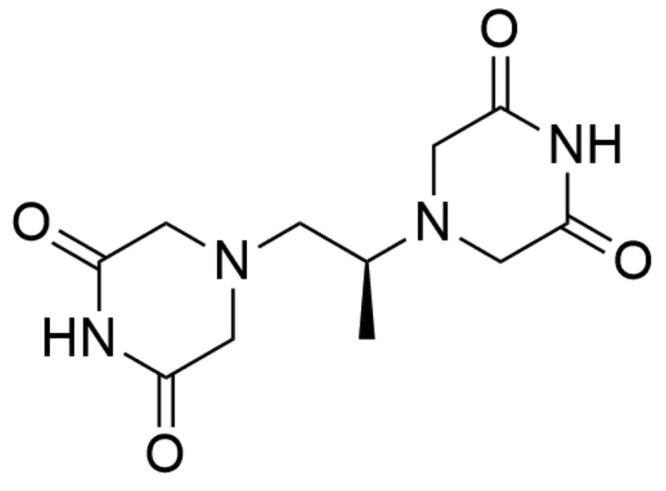
Chemical structure of dexrazoxane.

Dexrazoxane (4-[(2*S*)-2–(3,5-dioxopiperazin-1-yl)propyl]piperazine-2,6-dione; ICRF-187) is an ethylenediaminetetraacetic acid (EDTA) derivative and belongs to the group of bisdioxopiperazine compounds^161^. To date, the ICRF-187 (Figure 13) is the only drug approved for the treatment of anthracycline-induced cardiotoxicity. Due to the fact that ICRF-187 is an EDTA derivative, the cardioprotective properties of this compound are attributed to its ability to chelate free iron and iron complexed by anthracyclines—this prevents the formation of cardiotoxic reactive oxygen species^198^^,^^199^. The bisdioxopiperazine derivative act by Top II arrestment in a specific point in the catalytic cycle^200^. The dexrazoxane mechanism of action consists of blocking DNA dependent hydrolysis of ATP by Top II which prevents the N-terminal clamp opening and consequently blocks the release of transported DNA segment^201^. The ICRF-187 molecule is comprised of two piperazinedione rings bounded by a monomethyl substituted ethanediyl linker. Interactions between the enzyme and the compound occur between the Top II “tyrosine –dome” and the two ICRF-187 piperazinodione rings. The “tyrosine dome” is located approximately parallel to the piperazinodione rings of ICRF-187. Each of the Gln-365 of the Top II protomer forms a hydrophobic bond with the hydrogen of the imide dexrazoxane moiety. Furthermore, the ICRF-187 methyl side-chain forms an additional hydrophobic π-methyl bond with Tyr-28 of one of the Top II protomers. Losing this hydrophobic interaction by removing the linker group, but also replacing it with a larger side-chain significantly reduces the compound’s ability to inhibit Top II activity^202^^,^^203^.

##### Thiobarbiturates

**Figure 14.**
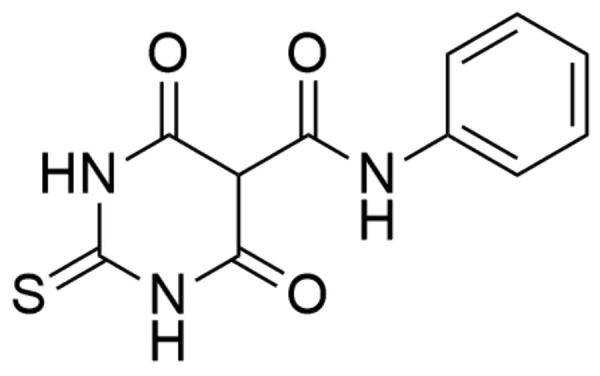
Chemical structure of merbarone.

Merbarone (5-[*N*-phenylcarboxamido]-2-thiobarbituric acid; NSC 336628), catalytic topoisomerase II inhibitor, is a thiobarbiturate analogue (Figure 14). In 1998 Fortune and Osheroff conducted biochemical studies and have classified the compound as a hTop IIα catalytic inhibitor^204^. Moreover, in 2012 Pastor *et al*. carried out research on AA8 ovary fibroblast Chinese hamster cell line which showed that merbarone is not only a cytotoxic but also genotoxic compound. Besides, studies reported the induction of the endoreduplication process by compound^205^. The detailed mechanism of hTop IIα inhibition remains still unknown and although it is not approved as a therapeutic agent. In the hTop IIα–DNA complex, Arg-804 and Tyr-805 are important elements that allows the transesterification reaction and splitting the DNA strand at the phosphate centre. The close proximity of the phosphorus atom of the DNA sugar-phosphate backbone and hydroxyl oxygen located in the Tyr-805 side-chain determines the hTop IIα catalysed reaction. Merbarone, creating a hydrogen bond between the oxygen of the amide residue and Tyr-805 of the topoisomerase, increases the distance between the phosphorus in the DNA backbone and the hydroxyl oxygen of the Tyr-805 side-chain. Scientists presume that by increasing the distance between Tyr-805 and the hTop IIα reaction site, merbarone prevents the 5′-phosphotyrosyl bond in the hTop IIα-DNA complex^206^. Currently, the drug is used as a tool for studies on Top II^191^.

## Conclusions and perspectives

DNA replication, transcription and repair mechanisms are crucial processes occurring in each cell and topoisomerases are one of the key elements of these processes. Due to their significant biological functions, enzyme structure and mechanisms of action, these enzymes have been one of the main molecular targets in the design of new anticancer agents for nearly 30 years. Despite all the collected data about topoisomerases, there are still some elements that are unclear for us. Now, a phenomenon that poses a great challenge to scientists around the world is the developing resistance to anti-topoisomerase drugs. The main challenge is to maintain topoisomerase sensitivity to drugs, e.g. by designing new enzymes or new drug delivery systems. Furthermore, studies on structure–activity relationship or molecular docking play an important role in the drug development process. The application of computational techniques or the use of special programs to study the interactions between individual structures enables scientists to identify the key elements of the compound structure responsible for their activity. Thanks to this it is possible to modify the structures of known compounds to improve their properties or to design and synthesise new derivatives based on computer simulation results. Development of new anti-topoisomerase drugs, e.g. may allow to limit the cardiotoxic effect of anthracyclines or reduce the incidence of drug-induced secondary cancers. After all, the growing importance and rapid development of molecular genetics and molecular biology may enable the use of anti-topoisomerase agents in personalised anticancer therapy.
